#  Unlocking the Power of AI: Healthcare Workforce Perception and Its Impact on their Work Performance in Saudi Arabia

**DOI:** 10.12669/pjms.41.3.11014

**Published:** 2025-03

**Authors:** Rakesh Kumar, Ajay Singh, Ahmed Subahi Ahmed Kassar, Mohammed Ismail Humaida, Sudhanshu Joshi, Manu Sharma

**Affiliations:** 1 Rakesh Kumar, PhD Department of Health Management, College of Public Health and Health Informatics, University of Hail, Saudi Arabia; 2 Ajay Singh, PhD Department of Management & Information Systems, College of Business Administration, University of Hail, Saudi Arabia; 3 Ahmed Subahi Ahmed Kassar, PhD Department of Public Health, College of Public Health and Health Informatics, University of Hail, Saudi Arabia; 4 Mohammed Ismail Humaida, PhD Department of Public Health, College of Public Health and Health Informatics, University of Hail, Saudi Arabia; 5 Sudhanshu Joshi, PhD School of Management, Doon University, Dehradun, Uttarakhand, India; 6 Manu Sharma, PhD Department of Management Studies, Graphic Era University, Dehradun, Uttarakhand, India

**Keywords:** Artificial Intelligence, Technology Readiness, Workforce Performance, Healthcare, Saudi Arabia

## Abstract

**Objective:**

To investigate the perception of AI among the healthcare workforce and its impact on their performance, with technology readiness acting as a moderating factor.

**Methods:**

In this cross-sectional study, a close-ended, self-administered survey questionnaire was used between 02 June to 04 August, 2024 to collect responses from 434 participants working in the public hospitals in Hail health cluster in Saudi Arabia. The study employed demographic summaries, descriptive statistics, regression analysis using Hayes’ Process, and regression diagnostics for data analysis. The data were analyzed using SPSS version 27.

**Results:**

The participant demographics indicated a majority of male respondents from the medical field, primarily aged between 36-45 years. Most participants had 9-10 or more years of experience in their current position and held graduate degrees in the healthcare sector of Saudi Arabia. Regression analysis using Hayes’ Process showed an insignificant negative impact of AI perception on workforce performance (β_1 = -0.0062, p = .315). However, technology readiness significantly moderated this effect, turning it into a positive and significant impact (β_3 = 0.2512, p = .0209).

**Conclusion:**

The study demonstrates that while AI perception alone has a negligible effect on workforce performance, its influence becomes significant when moderated by higher levels of technology readiness. Future research should examine how factors such as organizational culture and resource availability influence AI perceptions in healthcare.

## INTRODUCTION

In the rapidly evolving healthcare landscape, technological advancements - particularly in artificial intelligence (AI) are redefining the roles and performance expectations of healthcare professionals. The integration of AI into healthcare systems holds the potential to significantly enhance operational efficiency.^1^-^2^ It also boosts workforce performance by automating tasks, supporting clinical decision-making, and streamlining administrative processes.^3^ However, the perception of AI among the healthcare workforce is crucial to its successful implementation. Workforce perceptions can greatly influence the adoption speed and effectiveness of AI-driven innovations, ultimately translating into improved healthcare performance.^4^-^5^

Historically, workforce performance has typically been attributed to human factors such as motivation, skill level, and integration into organizational culture.^6^-^8^ However, with the growing adoption of AI, perceptions of technology, in addition to its ease of use, have become equally important factors.^1^-^3^ AI creates a highly enabling environment that enhances workforce performance when positive perceptions of it are paired with a high level of technology readiness.^9^-^11^ Conversely, negative perceptions of AI may discourage physicians from fully embracing its deployment in healthcare systems.^12^-^14^

There is a myriad of studies on AI adoption in the healthcare setting in Europe and North America.^3^-^5^,^8^-^10^ Precisely, however, little research has been conducted on the perception of AI in the healthcare workforce in Saudi Arabia.^11^,^12^-^14^ This mismatch is even more critical considering the Saudi Arabia’s visionary approach toward the year 2030, which strongly emphasizes the need for complete digital transformation and integration of AI into its health system.^15^ Only by understanding how the healthcare workforce’s views about AI influence performance on the ground will its adoption into healthcare translate into better outcomes.

The key focus of this study is to explore how perceptions about AI impact workforce performance in healthcare, with particular attention to the moderating role of technology readiness. These findings would, therefore, add to the literature on the adoption of AI in the health sector and provide insights to guide health organizations’ integration of AI into their operations.

## METHODS

The study participants were the working healthcare workforce from the public hospitals in Hail health cluster in Saudi Arabia. It includes workforce related to Medicine, Nursing, Allied Health Services, Administration, and others. In this cross-sectional study, the data was collected using a close-ended self-administrative survey questionnaire between 02 June to 04 August, 2024.

### Ethical Consideration:

Ethical approval for the study was obtained from the Ethical Review Committee, University of Hail; Dated: 27/05/2024, no. of research: H-2024-351.

### Informed Consent:

The participants provided informed consent after willingness to participate in the study.

### Inclusion and Exclusion Criteria:

The study considered the participants with a willingness to participate voluntarily. The unwilling participants were excluded.

### Instrument and variables:

The instrument used for collecting data in this study is a close-ended self-administrative survey questionnaire. The questionnaire includes one introductory part which contains statements about the objective of the study, obtaining informed consent, and maintaining the confidentiality of participants while responding to this questionnaire. Further, the questionnaire has four sections. The first section contains demographic details of the participants, like their gender, age, education, discipline, and experience in their current position. The second section contains the 11 items related to AI perception in healthcare.^16^ These items include the statements like “I believe that the use of AI in my specialty could improve the delivery of patient care” as an indicator for AI perception in healthcare.

Similarly, the third section contains 11 items related to workforce performance in healthcare.^17^ These items include the statements like “I have the competencies that my job requires” as an indicator for workforce performance in healthcare. Additionally, the fourth section contain 16 items related to Technology Readiness in healthcare.^18^ These items include the statements like “New technologies contribute to a better quality of life” as an indicator for Technology Readiness in Healthcare. A 5-point Likert type scale (Strongly disagree to Strongly Agree) was used to record the participant’s responses in this study. For the ease of participants, an English and Arabic version of the questionnaire was provided. Availability of questionnaire into English and Arabic version ensures the clarity and understanding of the questionnaire by respondents in the healthcare sector of Saudi Arabia.

### Sampling Approach and Data Collection:

The study included all the workforce from the selected health cluster in Saudi Arabia to respond to the survey questionnaire. The study required to calculate the minimum sample based on a number of assumptions. The first assumption was that 90% of the participants had a good understanding of AI in healthcare using a margin of error of 5% and a confidence interval of 95%. Therefore, the minimum sample required was 410 participants.

### Methods of Estimation and Analysis:

The collected data was analyzed statistically using SPSS version 27. The analysis includes demographic summary, descriptive statistics, regression analysis including the Hayes Process for moderation, and regression diagnostics. The descriptive summary indicates the frequency and percentage of different categories of characteristics provided by respondents, such as age, gender, education, experience, discipline, etc. The descriptive statistics indicate the minimum, maximum, mean, standard deviations, and Cronbach alpha for the variables of the study, like AI perception, workforce performance, and technology readiness. The regression by Hayes Process indicates the impact of AI perception on workforce performance, including the moderation impact of technology readiness in the healthcare sector of Saudi Arabia. Finally, the diagnostic includes regression assumption testing, such as normality, heteroscedasticity, independence of errors, and multicollinearity.

## RESULTS

The descriptive analysis indicates that 61.3% participants are male, and 38.7% participants are female. (Table-I). Furthermore, a major part of these respondents represents medicine discipline. Moreover, 41.9% of the participants represent an age category of 36-45 years. Additionally, one-fourth of the respondents had 9-10 years or more experience in their current position of healthcare in Saudi Arabia. Finally, more than half of the respondents have a graduation degree as a part of their education level (Table-I). The initial results reveal that AI perception in Healthcare (AIH) has a low mean score of 2.17, indicating a generally negative view among respondents. Furthermore, Workforce Performance (WP) and Technology Readiness (TR) are rated positively, with a means of 4.24 and 3.91, respectively. AIH shows the slightest variation in responses, while TR shows the most. All constructs have good reliability, with Cronbach’s Alpha values of 0.729 for AIH, 0.892 for WP, and 0.88 for TR (Table-II). The result indicates that among healthcare workforce, AI perception is low, but workforce performance and technology readiness are high.

**Table-I:** Descriptive Statistics.

Characteristic	Categories	N	%
Gender	Male	266	61.3%
	Female	168	38.7%
Discipline	Medicine	175	40.3%
	Nursing	105	24.2%
	Allied health	49	11.3%
	Administration	91	21.0%
	Others	14	3.2%
Age (in years)	18-25	42	9.7%
	26-35	112	25.8%
	36-45	182	41.9%
	46-55	70	16.1%
	> 55	28	6.5%
Experience in current position (Years)	0-2	42	9.70%
	3-4	28	6.50%
	5-6	49	11.30%
	7-8	98	22.60%
	9-10	112	25.80%
	> 10	105	24.20%
Education	Diploma	105	24.2%
	Graduate	245	56.5%
	Masters	84	19.4%

**Table-II:** Constructs and their Reliability.

Constructs	N	Min	Max	Mean	Std. Deviation	Cronbach Alpha
AIH	434	1.00	5.00	2.17	0.35	0.729
WP	434	1.00	5.00	4.24	0.40	0.892
TR	434	1.00	5.00	3.91	0.49	0.88

***Note:*** AIH = AI perception in Healthcare, TR = Technology Readiness, WP = Workforce Performance.

Further, the results of assumption tests for the regression analysis are calculated (Table-III). The Shapiro-Wilk test was used to check the normality of residuals, with a non-significant result (*W* = .958, *p* = .0659), indicating that the residuals are normally distributed. Additionally, the Durbin-Watson statistic of 2.290 suggests that the residuals are independent, confirming the assumption of independence of errors. Moreover, the Breusch-Pagan test for homoscedasticity yields a non-significant result (χ² = 3.203, *p* = .064), indicating that the residuals are homoscedastic. Lastly, the Variance Inflation Factor (VIF) value of 1.029 is below the threshold of five, indicating that multicollinearity is absent among the predictors.

**Table-III:** Regression Diagnostics Test.

Assumption	Test	Test Statistics	P-Value	Decision
Normality of Errors	Shapiro Wilk	.958	0.0659	Residuals are Normal
Independence of Errors	Durbin Watson	2.290	-	Residuals are Independent
Homoscedasticity	Breusch Pagan	3.203	0.064	Residuals are Homoscedastic
Collinearity	VIF	1.029	-	Multicollinearity is absent

Therefore, all key assumptions of the regression analysis are met. Finally, the results of a regression analysis conducted using the Hayes Process to examine the effect of AIH, TR, and their interaction (AIH*TR) on WP (Table-IV). The direct effect of AIH on WP is non-significant ( = -0.0062, = 0.0582, = -0.107, *p* = .315). However, TR significantly and positively influences workforce performance ( = 0.1486, = 0.0396, = 3.753, *p* < .001). The interaction term, AIH*TR, also significantly predicts workforce performance ( = 0.2512, = 0.1181, = 2.127, *p* = .0209). It indicates that the relationship between AIH and workforce performance is moderated by technology readiness. Moreover, the R^2^ value before including the interaction term is 0.0754. Further, after introducing the moderation effect, it increases to 0.2886, with an R^2^ change of 0.2132, suggesting that the interaction explains an additional 21.32% of the variance in WP. The overall model fit is significant, *F* (3, 430) = 5.287, *p* = .0014, indicating that the model significantly predicts WP.

**Table-IV:** Result of Regression Analysis using Hayes Process.

Variables	Coefficients	SE	T-value	P-value
AIH	-0.0062	0.0582	-0.10653	0.3155
TR	0.1486	0.0396	3.752525	0.0002
AIH*TR	0.2512	0.1181	2.127011	0.0209
Constant	4.2385	0.0193	219.6114	0
R-Square (Without Interaction Term)				0.0754
R-square (After Moderation)	-	-	-	0.2886
R-Square Change	-	-	-	0.2132
F-Value	-	-	-	5.2873
Model Fit (P-value)	-	-	-	0.0014

***Note:*** AIH = AI perception in Healthcare, TR = Technology Readiness.

## DISCUSSION

The findings of this study revealed that employees with high level of technology readiness have positive perception towards the use of AI and it has significant effect on workforce performance. The findings further indicate that alone the perception of AI in healthcare (AIH) found a low influence on workforce performance, which contrasts with previous research suggesting a more positive impact of AI on healthcare outcomes.^14^,^19^-^20^ However, with a moderating role, technology readiness (TR) was found to significantly enhance workforce performance, consistent with other studies that highlight the importance of technological preparedness in driving organizational success.^18^,^21^-^22^ The interaction between AIH and TR was also significant, suggesting that while AI perception alone does not strongly impact workforce performance, it becomes more influential when technology readiness is high. These results align with existing research emphasizing the role of moderating factors, such as technological infrastructure, in maximizing the benefits of AI adoption in healthcare.^1^-^2^,^5^-^8^

This study expands the existing literature by focusing on the healthcare workforce in Saudi Arabia, where AI adoption in healthcare is still in its early stages.^15^,^23^ The findings underscore the importance of improving workforce technology readiness to leverage AI’s potential better. Most previous studies have targeted either government institutions, private firms, or educational settings.^7^,^9^-^11^,^18^,^21^-^22^ However, the present study focused on health sector, adding new insights into how perceptions of AI and technology readiness influence workforce performance in this critical sector with the rapid development of healthcare technologies. Hence, training and development programs to enhance AI literacy and technological readiness among health professionals urgently need to be considered.^16^,^24^-^25^ From the results, male respondents were dominant in the research sample; most of the respondents belonged to the Medicine discipline. Most respondents were middle-aged and had been experienced in their current professions for several years as well. Also, over half of the respondents had a graduation degree; thus, it is relatively educated. While these demographic characteristics were part of the study, no significant associations were noted with AI perception or workforce performance, which concurs with literature showing that demographic factors may be less critical to AI integration. Nevertheless, other contextual factors that could affect employee perception towards AI, such as organizational culture and resource availability, should be studied in future research.

### Limitations:

Several limitations exist in this study. It focused on just one health cluster within the Saudi Arabian healthcare sector and thus might need further replication to generalize outcomes in other health cluster also. Also, relying on self-reported measures for AI perception and workforce performance could result in self-report bias. In light of this consideration, future research should involve more diversified populations while specializing in objective performance metrics to establish better generalizability.

## CONCLUSION

This study shows that technology readiness plays an essential role in improving workforce performance when considering AI perception in Saudi Arabian health care. It is manifested that AI perception alone presents a somewhat limited effect, but its power increases substantially upon being complemented by higher levels of technology readiness. Similarly, the Medicine discipline requires tailored strategies in the integration of AI. Again, in the absence of significant associations of demographic factors, gender and professional background are two variables that may influence perceptions about AI in healthcare and need further investigation. Fostering a tech-ready workforce is essential for maximizing AI’s benefits in healthcare settings.
